# Advancing Chikungunya Prevention and Vaccination in Mexico: A Position Paper by the Immunization Committee of the Mexican Association of Pediatric Infectious Diseases

**DOI:** 10.3390/vaccines14090756

**Published:** 2026-08-30

**Authors:** Jorge A. Vázquez-Narváez, Martha Avilés-Robles, Carmen Espinosa-Sotero, Cesar Adrián Martínez-Longoria, Sarbelio Moreno-Espinosa, Monica L. Reyes-Berlanga, Enrique Chacon-Cruz

**Affiliations:** 1 Department of Epidemiology and Pediatrics, Hospital Ángeles Morelia, Morelia 58350, Mexico; 2 Department of Infectious Diseases, Hospital Infantil de México Federico Gómez, Mexico City 06720, Mexico; marmtom@hotmail.com; 3 Department of Pediatrics, Hospital General de México “Dr. Eduardo Liceaga”, Mexico City 06726, Mexico; carmenespinosa6@hotmail.com; 4 School of Medicine and Health Sciences, Tecnológico de Monterrey (TecSalud), Monterrey 64700, Mexico; ceadml@hotmail.com; 5 Infectious Diseases Section, Department of Pediatrics, Hospital Médica Sur, Mexico City 14050, Mexico; sarbelio.infecto@gmail.com; 6 Infectious Diseases Section, Hospital MAC Irapuato, Irapuato 36520, Mexico; reyesberlanga@yahoo.com.mx; 7 Department of Education and Research, Think Vaccines LLC, Houston, TX 77005, USA; enrique.chacon@thinkvaccines.org

**Keywords:** chikungunya, chikungunya vaccines, arboviruses, Mexico, public health, immunization policy

## Abstract

**Background:** Chikungunya virus (CHIKV) is a mosquito-borne alphavirus transmitted predominantly by *Aedes aegypti* and *Aedes albopictus*. Infection is characterized by an acute febrile illness accompanied by debilitating polyarthralgia, myalgia, and cutaneous manifestations. Although the acute syndrome is usually self-limited, a substantial proportion of patients experience persistent musculoskeletal symptoms that may progress to chronic inflammatory arthritis, resulting in prolonged disability and impaired quality of life. **Objective:** To summarize current evidence on the epidemiology, virology, immunopathogenesis, clinical manifestations, laboratory diagnosis, prevention, and vaccine development of CHIKV, with particular emphasis on its implications for public health policy and immunization strategies in Mexico. **Methods:** This position paper was developed through a structured narrative review of the scientific literature. Publications indexed in PubMed, Scopus, and Embase, together with documents issued by the World Health Organization (WHO), Pan American Health Organization (PAHO), Centers for Disease Control and Prevention (CDC), U.S. Food and Drug Administration (FDA), and European Medicines Agency (EMA), were critically reviewed. Evidence was selected according to its scientific quality and relevance to Mexico and Latin America. Because this work represents an expert consensus rather than a systematic review, no formal risk-of-bias assessment was performed. **Results:** CHIKV circulates through both urban and sylvatic transmission cycles and continues to expand into regions where competent mosquito vectors are established. Disease progression is driven by complex innate and adaptive immune responses, with exaggerated inflammatory activation contributing to chronic rheumatologic sequelae. Laboratory confirmation relies primarily on molecular assays during the viremic phase and serological testing thereafter. Recent advances in vaccine development—including live-attenuated and virus-like particle (VLP) platforms—have demonstrated favorable immunogenicity and acceptable safety profiles. However, vaccination should be considered one component of an integrated prevention strategy that also includes vector surveillance, environmental control, and rapid outbreak detection. **Conclusions:** Chikungunya remains an emerging global public health challenge because of its expanding geographic distribution, epidemic potential, and long-term clinical consequences. Incorporating vaccination into comprehensive arboviral control programs, together with strengthened surveillance and integrated vector management, may substantially reduce disease burden. This position paper provides evidence-based recommendations to support future chikungunya prevention and vaccination policies in Mexico.

## 1. Introduction

Chikungunya virus (CHIKV), a positive-sense RNA member of the genus *Alphavirus* and family *Togaviridae*, is transmitted to humans mainly by infected *Aedes* aegypti and *Aedes* albopictus mosquitoes. The typical illness begins abruptly with fever, marked polyarthralgia, myalgia, and a maculopapular eruption. Although acute symptoms often subside within one or two weeks, recovery may be incomplete: some patients experience inflammatory musculoskeletal complaints for months or years, with consequent chronic pain, loss of function, and deterioration in health-related quality of life [1].

CHIKV was first isolated during the 1952–1953 outbreak in present-day Tanzania. It is no longer confined to a limited geographic area and has caused repeated epidemics across Africa, Asia, Europe, and the Americas, with reports from more than 100 countries. Its spread has been favored by the widening range of competent Aedes vectors, international mobility, rapid urban growth, and climate-related environmental change, which together increase the likelihood of introduction and amplification in previously unaffected settings [2].

Mortality attributable to chikungunya is generally low, yet the overall burden is considerable. Roughly three out of four infected persons develop clinical disease, and a meaningful proportion later report prolonged arthralgia or inflammatory arthritis. These persistent manifestations translate into disability, repeated use of health services, and important social and economic consequences [2].

Historically, prevention depended on mosquito control and individual measures to avoid bites. Continued transmission and recurrent outbreaks in endemic areas, despite these interventions, demonstrate the limits of relying on vector control alone. The licensing of chikungunya vaccines creates an opportunity to add immunization to existing prevention programs and thereby lessen disease burden [3].

The growing geographic reach of CHIKV and the availability of licensed vaccines make a current synthesis particularly relevant. This position paper integrates evidence on viral biology, epidemiology, immune mechanisms, clinical disease, diagnosis, management, prevention, and vaccination, and considers how these findings may inform immunization policy in Mexico.

## 2. Methods

This position paper was developed through a structured narrative review. PubMed, Scopus, and Embase were searched from database inception through 13 May 2026. Search concepts combined controlled vocabulary and free-text terms for ‘chikungunya’ or ‘CHIKV’ with ‘epidemiology’, ‘Mexico’, ‘Latin America’, ‘pathogenesis’, ‘immunopathogenesis’, ‘clinical manifestations’, ‘diagnosis’, ‘treatment’, ‘prevention’, ‘vaccine’, ‘vaccination’, ‘surveillance’, ‘forecasting’, ‘economic burden’, and ‘cost’. Reference lists of key reviews were screened, and official documents from WHO, PAHO, CDC, FDA, EMA, and Mexican public health authorities were consulted. English- and Spanish-language sources were eligible; no language was intentionally excluded when a reliable translation could be assessed.

Evidence was selected for relevance to the prespecified domains and applicability to Mexico or Latin America. Priority was given to systematic reviews, randomized and non-randomized clinical studies, epidemiological and modeling studies, primary vaccine trials, pharmacovigilance reports, and current regulatory or public health guidance. The authors synthesized findings narratively, emphasizing consistency across sources, recency, methodological strength, and direct regional relevance. Draft recommendations were discussed by the Immunization Committee, revised iteratively, and retained when consensus was achieved among participating authors.

Because the objective was an expert position paper rather than a systematic review, no protocol was registered, no duplicate screening log was maintained, no quantitative meta-analysis was performed, and no formal risk-of-bias instrument was applied. Consequently, the total number of records identified and excluded cannot be reconstructed reliably, and a PRISMA flow diagram would imply a level of systematic-review methodology that was not used. These limitations reduce reproducibility and are acknowledged explicitly.

## 3. Rationale

The resurgence and continuing geographic spread of CHIKV show why prevention must extend beyond conventional mosquito-control activities. After the virus reached the Americas in 2013, successive epidemics affected millions of people and produced substantial clinical, economic, and public health consequences in tropical and subtropical areas. Because *Aedes* mosquitoes are widely established and population susceptibility persists amid urbanization and increasing travel, additional outbreaks remain likely [3].

Licensure of the first chikungunya vaccines has changed the prevention landscape. Development programs have examined live-attenuated, inactivated, and virus-like particle (VLP) approaches, several of which generated strong immune responses with acceptable safety findings. Phase 3 evaluation of a live-attenuated candidate, for example, documented high seroresponse after one dose and sustained neutralizing antibodies, supporting subsequent regulatory authorization for selected populations [4].

Moving from licensure to broad public health use nevertheless requires resolution of several issues: which groups should be prioritized, whether vaccination should be based on exposure risk, how protection and safety evolve over time, and how immunization can be integrated into established programs. WHO, CDC, and national and international advisory bodies are therefore reviewing the evidence to guide recommendations [5,6,7].

Against this rapidly changing background, the present paper evaluates vaccine technologies, immune responses, efficacy-related evidence, safety, and implementation considerations, with emphasis on their relevance to vaccination policy and public health decisions in Mexico.

## 4. Virus Characteristics

Chikungunya virus (CHIKV) is an enveloped, positive-sense single-stranded RNA virus belonging to the genus Alphavirus within the family Togaviridae. Mature virions measure approximately 60–70 nm in diameter and display an icosahedral nucleocapsid enclosed by a host-derived lipid bilayer. Embedded within this envelope are approximately 80 trimeric glycoprotein spikes that project from the virion surface and play essential roles in host-cell recognition and viral entry. The viral genome is approximately 11.8 kb in length and contains both a 5′ methylated cap and a 3′ polyadenylated tail, structural features required for efficient translation and replication within infected cells [8,9].

Two large open reading frames organize the CHIKV genome. The 5-prime region produces the four non-structural proteins nsP1 through nsP4, which form the machinery for viral RNA synthesis and transcription. The 3-prime region encodes a structural polyprotein that is cleaved into capsid and the envelope-related components E3, E2, 6K/transframe (TF), and E1. Together, these products package genomic RNA, assemble viral particles, and generate mature infectious virions [10].

E1 and E2 are especially important for viral biology and vaccine-induced immunity because antibodies that neutralize CHIKV predominantly recognize these surface glycoproteins. E1–E2 heterodimers form trimeric spikes on the virion. E2 is chiefly responsible for receptor engagement and attachment, whereas the fusion peptide in E1 mediates membrane fusion after endosomal acidification, permitting cytoplasmic release of the nucleocapsid and initiation of replication [8,11].

Structural studies identify matrix-remodeling associated protein 8 (MXRA8) as a principal entry receptor for CHIKV. Each trimeric E1-E2 spike can engage three MXRA8 molecules through contacts with the receptor’s D1 and D2 domains. This multivalent binding strengthens attachment and favors internalization; it may also help explain the virus’s predilection for musculoskeletal tissues [12,13,14].

## 5. Pathophysiology

After deposition by an infected *Aedes* mosquito, CHIKV replicates rapidly near the inoculation site, producing high viremia and activating innate and adaptive immunity. Entry involves E2 interactions with cellular glycosaminoglycans and engagement of MXRA8, a major CHIKV receptor. These events permit internalization and establish infection after an incubation interval that is usually 2–12 days [13,14,15].

Initial infection involves skin-associated cells, including fibroblasts, keratinocytes, endothelial cells, and resident macrophages. The incoming positive-sense RNA is translated to produce replication-associated non-structural proteins, while subgenomic RNA supplies structural proteins for virion assembly and release. Abundant MXRA8 on synovial fibroblasts, chondrocytes, and related cells may account in part for joint tropism and the link between acute infection and later rheumatologic disease [16,17].

Clinical severity reflects not only viral injury but also the intensity and duration of the host inflammatory response. Infection induces cytokines and chemokines that recruit monocytes, macrophages, neutrophils, and lymphocytes. Although this response contributes to viral control, dysregulated or persistent activation is associated with severe presentations and continuing synovial inflammation; particularly high mediator concentrations have been described in fatal and severe cases [13,18].

Vascular dysfunction may accompany CHIKV infection through direct endothelial involvement and inflammation-driven injury. Excess reactive oxygen species and superoxide promote oxidative damage, and inflammatory signaling increases adhesion molecules such as ICAM-1, enhancing endothelial activation and leukocyte recruitment. Upregulation of inducible nitric oxide synthase and the resulting nitric oxide production can further impair barrier integrity [19].

Matrix metalloproteinase upregulation provides another pathway to severe disease by degrading extracellular matrix and disrupting endothelial junctions. The resulting permeability can manifest as edema, plasma leakage, or petechiae and, rarely, hemorrhage, hypotension, or shock. Loss of barrier function may also facilitate dissemination beyond the initial site. Acute laboratory findings can include inflammatory-marker elevation, high cytokine concentrations, mild thrombocytopenia, and transient coagulation changes. Recovery of infectious virus or viral RNA from blood, saliva, urine, and cerebrospinal fluid further illustrates the systemic distribution of infection [13].

## 6. Epidemiology of Chikungunya Virus

Chikungunya virus (CHIKV) is an emerging mosquito-borne alphavirus with sustained transmission across tropical and subtropical regions of Africa, Asia, the Indian Ocean islands, and, more recently, the Americas. Since its initial isolation during an outbreak of acute febrile illness in Tanzania in 1952–1953, CHIKV has evolved from a geographically restricted pathogen into a global public health concern. Historical descriptions of dengue-like febrile illnesses dating back to the eighteenth and nineteenth centuries suggest that chikungunya outbreaks probably occurred long before the virus was formally identified, although they were likely misclassified because of the clinical similarities between these arboviral infections.

The standard epidemiologic classification recognizes the West African, East/Central/South African (ECSA), and Asian genotypes; the Indian Ocean lineage (IOL) emerged from the ECSA genotype. The Asian genotype arose after spreading into Southeast Asia. Endemic circulation in Africa produces sporadic disease and periodic epidemics, while the 1958 Bangkok outbreak is commonly cited as an early well-documented event outside Africa [8].

*Aedes* aegypti and *Aedes* albopictus account for most transmission, although other *Aedes* species and occasional Culex involvement have been described in sylvatic settings. In the African sylvatic cycle, the virus circulates between forest mosquitoes, non-human primates, and other vertebrates, with intermittent human spillover. Epidemic urban transmission instead follows a human-mosquito-human pattern, usually dominated by *Aedes* aegypti. Seasonal rainfall and vector density shape this cycle, with transmission often peaking earlier in the year in the Southern Hemisphere and later in Mexico, Central America, and the Caribbean [20,21].

Although considerable progress has been made in understanding CHIKV ecology, predicting epidemic activity remains challenging. Historical epidemiological observations suggest recurrent regional epidemics approximately every 7–20 years and larger global epidemic waves every 40–50 years. These cyclic patterns are thought to reflect complex interactions among viral evolution, environmental conditions, vector density, climatic variability, and the gradual accumulation or loss of population immunity [22].

During the last two decades, CHIKV has achieved an extensive global distribution. Local transmission has been documented in more than 119 countries and territories spanning all six WHO regions, and competent *Aedes* aegypti populations occur in additional areas without recognized transmission. Viral adaptation to *Aedes* albopictus, travel, urban expansion, globalization, and climate change have accelerated this spread. More than 10 million cases have been reported, and approximately 1.3 billion people are estimated to live in areas where transmission is possible [23,24].

Before the twenty-first century, most outbreaks remained geographically restricted to Africa and Asia. Since 2004, however, epidemic activity has intensified considerably. The outbreak that originated in Kenya and subsequently spread throughout islands of the Indian Ocean—including Réunion, Mauritius, Seychelles, Madagascar, and Mayotte—represented a major turning point in the global epidemiology of CHIKV. Serological investigations performed shortly after the epidemic demonstrated exceptionally high attack rates, with seroprevalence approaching 75% in some affected communities, illustrating the remarkable transmissibility of the virus under favorable ecological conditions [25].

Europe has also experienced the consequences of increasing international travel mobility. Imported infections have been reported since 2006 among travelers returning from endemic regions, and in 2007 Italy documented the first locally acquired outbreak of chikungunya in Europe, confirming that autochthonous transmission can occur wherever competent mosquito vectors become established [26].

A major epidemiological shift followed the introduction of CHIKV into the Americas in late 2013. Rapid dissemination was facilitated by widespread Aedes populations and immunologically naïve human populations. The Asian genotype predominated during the initial epidemics, whereas Brazil subsequently experienced sustained circulation of the ECSA genotype. The ECSA-derived Indian Ocean lineage (IOL) should be distinguished from the broader ECSA genotype when sequence data support that assignment [23].

According to the Pan American Health Organization (PAHO), nearly 1.8 million suspected chikungunya cases were reported across the Americas during the first two epidemic years (2014–2015). Although annual reported case numbers subsequently declined, transmission resurged in 2023, when more than 411,000 suspected cases and 515 deaths were reported. During 2024, approximately 428,000 additional cases and 213 deaths were documented, coinciding with the simultaneous resurgence of dengue throughout the region [27].

In Mexico, an imported infection was identified in Jalisco in May 2014, followed later that year by the first locally acquired case in Chiapas. Molecular monitoring showed predominance of the Asian genotype during the early epidemic. Transmission reached its national maximum in 2015, when surveillance recorded more than 12,500 confirmed autochthonous cases, chiefly from May through August. Reporting has fallen markedly since 2019, with sporadic annual cases and a persistent concentration in southern and coastal states. Adults aged 25–44 years have been most frequently affected [28].

Recent national activity has been limited, but focal transmission has not disappeared. A Chiapas outbreak in 2015 included 67 laboratory-confirmed infections, mainly among persons aged 10–59 years, and involved an Asian-lineage virus transmitted by *Aedes* aegypti [29]. In Guerrero, serologic evaluation of febrile patients found markers of previous infection in 28.3% and findings consistent with acute infection in 11% [30].

The continuing geographic expansion of CHIKV, its capacity to produce explosive epidemics, and the considerable burden associated with chronic musculoskeletal disease have positioned chikungunya among the priority pathogens for vaccine research and development. In recognition of its epidemic potential, the Coalition for Epidemic Preparedness Innovations (CEPI) incorporated CHIKV into its priority portfolio in 2018. This prioritization has accelerated global investment in vaccine research, ultimately leading to the recent regulatory approval of the first chikungunya vaccine, reinforcing international efforts to accelerate vaccine development and improve global preparedness for future outbreaks.

## 7. Clinical Presentation and Disease Course

Clinical manifestations of chikungunya virus (CHIKV) infection evolve through three clinically recognized, although overlapping, phases: the acute, post-acute, and chronic phases. Although the duration and severity of symptoms vary among individuals, this classification provides a useful clinical framework for understanding disease progression and guiding patient management [31,32,33,34].

### 7.1. Acute Phase

The acute phase typically lasts up to two weeks following symptom onset and is characterized by the abrupt development of high fever accompanied by intense, frequently disabling polyarthralgia. Joint pain is typically bilateral and symmetrical, predominantly involving the wrists, ankles, hands, and feet, although larger joints may also be affected. Additional manifestations commonly include myalgia, headache, fatigue, and a maculopapular rash that generally develops during the first week of illness [35].

Although systemic manifestations usually improve within several days, the intensity of the inflammatory response during the acute stage appears to influence long-term outcomes. Patients presenting with severe polyarthralgia or polyarthritis or marked inflammatory activity are more likely to experience persistent musculoskeletal symptoms after resolution of the acute infection [36]. Less common but clinically important complications have also been reported, including neurological, cardiovascular, hepatic, renal, and ocular involvement, particularly among neonates, older adults, and individuals with underlying medical conditions [37,38,39].

### 7.2. Post-Acute Phase

The post-acute period extends approximately from the second week through the third month after symptom onset. During this phase, joint pain may recur or persist and can be accompanied by morning stiffness, tenosynovitis, or inflammatory arthritis. Symptoms often vary over time. Continued immune-mediated inflammation after viremia resolves may make this interval a bridge between acute disease and chronic rheumatologic manifestations in susceptible patients [40,41].

### 7.3. Chronic Phase

The chronic phase is defined by the persistence or recurrence of articular manifestations for more than three months after the initial infection. Chronic chikungunya arthritis may persist for months or even years and is believed to result primarily from sustained immune activation, although persistent viral RNA and viral antigens have been detected in synovial and musculoskeletal tissues in some patients. Persistent synovitis, inflammatory arthritis, chronic arthralgia, fatigue, and functional impairment represent the principal long-term consequences of infection and account for a substantial proportion of the overall disease burden. These chronic manifestations significantly affect quality of life, daily activities, and healthcare utilization, reinforcing the importance of effective preventive measures, including vaccination [40,41]. The persistence of chronic rheumatologic manifestations represents the principal rationale for preventive vaccination, as no specific antiviral therapy is currently available to prevent progression once infection has occurred.

### 7.4. Immunopathogenesis and Implications for Vaccine Development

During the acute phase of chikungunya virus (CHIKV) infection, high levels of viremia are accompanied by robust activation of the innate immune response, characterized by increased expression of proinflammatory mediators such as IL-6, IL-8, IFN-α, IP-10, MCP-1, and MIG. Multiple studies have demonstrated associations between elevated levels of IL-1β, IL-17A, IL-27, and granulocyte–macrophage colony-stimulating factor (GM-CSF) and the severity of acute joint manifestations. Type I interferon signaling plays a critical role in viral control, and impaired interferon responses have been associated with more severe disease phenotypes [42,43].

In the chronic phase, accumulating evidence suggests that persistent inflammation and immune activation may continue despite the absence of detectable infectious virus in synovial fluid. Viral RNA and viral antigens have been identified in synovial macrophages months after acute infection, together with infiltration by natural killer cells and CD4+ T lymphocytes. Although the prevalence of chronic manifestations gradually declines over time, a substantial proportion of patients continue to experience persistent joint pain long after the acute phase of infection. High viral loads and intense inflammatory responses during the acute phase have been consistently identified as predictors of chronic arthralgia. In this context, elevated levels of IL-6, GM-CSF, TNF-α, IL-1β, IL-5, and IL-12 during early infection have been proposed as potential immunological biomarkers associated with progression toward chronic disease [44,45,46]. These immunopathogenic mechanisms provide the biological rationale for vaccine-induced neutralizing antibody responses aimed at preventing viremia, limiting inflammatory activation, and reducing the risk of chronic musculoskeletal sequelae.

### 7.5. Clinical Outcomes and Immune Protection

Although most CHIKV infections are self-limited, severe clinical manifestations and fatalities may occur, particularly among neonates, older adults, pregnant women, and individuals with underlying medical conditions. Reported complications include neurological disorders, myocarditis, cardiac dysfunction, acute kidney injury, and, less frequently, multiorgan involvement, emphasizing that chikungunya is not exclusively a musculoskeletal disease [44].

The humoral immune response develops rapidly following infection. CHIKV-specific IgM antibodies become detectable within the first days after symptom onset and are subsequently followed by the production of high-affinity neutralizing IgG antibodies, predominantly of the IgG3 subclass. These antibodies play a central role in viral clearance and are considered the principal correlate of long-term protective immunity against reinfection [45].

Natural infection generally produces long-lasting immune memory and protection from reinfection with homologous strains. However, durable antiviral immunity does not preclude chronic inflammatory symptoms. The coexistence of viral clearance with persistent musculoskeletal disease illustrates why a successful vaccine should induce strong neutralizing responses without promoting prolonged or excessive immune activation [46].

These observations have important implications for vaccine development. An ideal CHIKV vaccine should not only generate durable protective immunity against infection but also reduce the incidence of chronic musculoskeletal complications that account for much of the long-term clinical and socioeconomic burden associated with chikungunya. This concept has guided the development of current vaccine candidates, which primarily aim to prevent symptomatic disease while potentially reducing the burden of chronic rheumatologic sequelae.

### 7.6. Diagnosis of Chikungunya Virus Infection

Accurate laboratory confirmation of chikungunya virus (CHIKV) infection depends primarily on the timing of specimen collection in relation to symptom onset. Current World Health Organization (WHO) recommendations describe three complementary diagnostic approaches: molecular detection of viral RNA, serological testing, and viral isolation. Selection of the appropriate diagnostic method should be guided by the stage of infection, as viral replication predominates during the early acute phase, whereas antibody-based assays become increasingly informative after seroconversion [47,48,49].

During the first week of illness, when viremia is highest, nucleic acid amplification techniques—particularly quantitative reverse-transcription polymerase chain reaction (RT-qPCR)—represent the preferred diagnostic method because of their high sensitivity and specificity. Viral isolation may also be performed during this period by inoculating clinical specimens into susceptible cell lines such as Vero, BHK-21, or HeLa cells, where characteristic cytopathic effects generally become evident within several days. Although viral isolation remains the virological reference standard for confirming viable virus, its routine clinical use is limited by prolonged turnaround times and the requirement for biosafety level 3 (BSL-3) laboratory facilities [50].

As viremia declines, serological assays become the cornerstone of laboratory diagnosis. Anti-CHIKV IgM antibodies are typically detectable between 3 and 8 days after symptom onset, may persist for several months, and in some individuals remain measurable for up to two years. CHIKV-specific IgG antibodies usually appear shortly thereafter, generally between days 4 and 10, and may persist for many years, reflecting previous infection and long-term humoral immunity. The enzyme-linked immunosorbent assay (ELISA) remains the most widely used serological platform for detecting both antibody classes because of its practicality and diagnostic performance [51].

Because no single laboratory method is optimal throughout the entire course of infection, diagnostic interpretation should always consider the patient’s clinical presentation and the interval between symptom onset and specimen collection. Molecular testing is recommended during the viremic phase, whereas serological assays are more appropriate once the humoral immune response has developed. This complementary diagnostic approach maximizes diagnostic accuracy and facilitates appropriate clinical management and epidemiological surveillance. In regions where dengue and Zika viruses co-circulate, multiplex molecular assays may improve diagnostic accuracy during the acute phase and facilitate appropriate public health surveillance.

Table 1 and Table 2 present the principal diagnostic methods, the stage of illness in which each is most informative, and the reported measures of diagnostic performance. Table 3 summarizes the clinical and diagnostic features used to distinguish chikungunya from dengue and Zika virus infection.

### 7.7. Treatment

To date, no antiviral drug has been approved specifically for CHIKV infection. Management is therefore supportive and focuses on symptom relief, hydration, prevention of complications, and preservation of function. Because polyarthralgia and myalgia often dominate the clinical picture, adequate analgesia is central to care. Education and psychological support may also benefit patients with prolonged or disabling symptoms [54].

Paracetamol (acetaminophen) is generally the first pharmacologic choice for fever and mild or moderate pain. Total exposure should be considered because excessive cumulative dosing and underlying liver disease increase the risk of hepatotoxicity. If pain remains inadequately controlled, tramadol, codeine, or another opioid may be considered according to clinical severity and individual risk.

The use of non-steroidal anti-inflammatory drugs (NSAIDs) during the acute phase of chikungunya remains controversial. Although several clinical guidelines support their use after dengue has been reasonably excluded clinically and/or laboratory testing has been performed, other recommendations advise caution because NSAIDs may increase the risk of bleeding, acute kidney injury, and other adverse events, particularly in patients with dehydration or unrecognized dengue coinfection. Therefore, exclusion of dengue should be considered before initiating NSAID therapy in regions where both arboviruses co-circulate.

Management should be tailored to age, comorbidities, complications, and overall disease severity. Because current therapy is supportive rather than antiviral, prevention remains especially important; vaccination and integrated vector management are complementary measures for reducing both individual illness and population-level burden.

### 7.8. Chronic Phase Management

Care of chronic chikungunya focuses on suppressing persistent inflammation, controlling pain, maintaining joint function, and protecting quality of life. Since musculoskeletal disease can continue long after acute infection, treatment should reflect the degree of synovitis, symptom intensity, and functional limitation.

Treatment options include analgesics, brief corticosteroid courses, disease-modifying antirheumatic drugs (DMARDs), and selected antimalarial agents such as hydroxychloroquine; chloroquine is now rarely recommended because of its limited efficacy and safety concerns. Among DMARDs, methotrexate has shown the most consistent benefit for persistent inflammatory arthritis, including improvements in pain and function. Carefully limited corticosteroid courses may help control inflammatory flares.

Follow-up should use validated measures when feasible. The Visual Analog Scale, Routine Assessment of Patient Index Data 3 (RAPID3), and Disease Activity Score-28 (DAS28) provide standardized assessment of pain, physical function, inflammatory activity, and patient-reported outcomes.

Although no antiviral therapy modifies chronic disease progression, early recognition and appropriate management of inflammatory arthritis may reduce long-term disability. In selected patients with severe acute polyarthritis, short courses of low-dose dexamethasone have been reported to decrease symptom severity and shorten recovery time when conventional analgesic therapy is insufficient. Given the absence of specific antiviral therapy, prevention through vaccination and integrated vector-control measures remains the most effective strategy to reduce the burden of severe chikungunya disease.

### 7.9. Severe Chikungunya Disease

A minority of CHIKV infections progress to severe disease, particularly in vulnerable hosts. Reported complications include respiratory failure, myocarditis, hemodynamic instability, hepatitis, acute kidney injury, bleeding, encephalitis, and encephalopathy. Admission is warranted for organ dysfunction, significant hemorrhage, unstable circulation, medically refractory pain, or decompensation of a pre-existing illness. Prompt multidisciplinary care is important to limit adverse outcomes.

## 8. Management in Vulnerable Populations

### 8.1. Pregnancy

Pregnant women should receive supportive care, with acetaminophen considered the preferred analgesic and antipyretic throughout pregnancy. Aspirin and non-steroidal anti-inflammatory drugs are generally avoided because of their potential fetal risks, including premature closure of the ductus arteriosus, impaired fetal renal function, and adverse pregnancy outcomes. Maternal and fetal monitoring is recommended, particularly during the third trimester. When maternal infection occurs during the highly viremic period immediately before delivery, delaying delivery, when clinically feasible, may reduce the risk of vertical transmission. Current evidence does not support cesarean delivery as an effective strategy to prevent mother-to-child transmission, and transmission through breastfeeding has not been demonstrated [55].

### 8.2. Older Adults and Patients with Comorbidities

People older than 60 years and those with chronic comorbidities have a higher probability of atypical or severe chikungunya and unfavorable outcomes. They require close observation and timely hospital referral when clinical deterioration or worsening of an underlying condition is suspected [55].

### 8.3. Pediatric Chikungunya

In children, chikungunya virus infection is generally a self-limiting illness with an excellent prognosis, although infants—particularly neonates—and children with neurological or hemodynamic complications are at greater risk of severe disease. Because no antiviral therapy is currently available, treatment remains supportive and focuses on maintaining adequate hydration, controlling fever and pain with acetaminophen, and monitoring for warning signs.

Most pediatric patients can be managed as outpatients. However, severe manifestations—including encephalitis, seizures, circulatory shock, respiratory failure, or multiorgan dysfunction—require hospitalization and, in some cases, intensive care support. Management may include hemodynamic stabilization, anticonvulsant therapy, respiratory support, and treatment of concomitant infections, including dengue or bacterial coinfections when present. Non-steroidal anti-inflammatory drugs should be introduced cautiously and only after dengue infection has been reasonably excluded [55].

Overall prognosis in children is favorable, with more than 90% achieving complete recovery and mortality remaining below 1%. Nevertheless, infants younger than one year are at increased risk of severe complications, including capillary leak syndrome, hyponatremia, shock, and multiorgan failure. Although long-term sequelae are uncommon, chronic arthralgia has been reported in approximately 9–16% of pediatric patients. Children with vertically acquired CHIKV infection or severe neurological involvement may subsequently develop developmental delay, language impairment, epilepsy, or other neurological sequelae requiring prolonged multidisciplinary follow-up [55]. The clinical pathway from symptom onset to disposition is summarized in Table 4.

## 9. Transmission, Risk Factors, and Prediction of Chikungunya Outbreaks

### 9.1. Transmission

The global reach of CHIKV is closely related to the ecological versatility of its vectors. *Aedes* aegypti and *Aedes* albopictus have become established across tropical and subtropical zones and increasingly in temperate regions. Their competence for transmission, geographic range, and adaptation to human environments have enabled repeated emergence and re-emergence of chikungunya [56,57,58].

Two epidemiologic cycles sustain the virus. Forest transmission in parts of Africa involves mosquitoes, non-human primates, and other vertebrate hosts and occasionally spills over to people. In most other settings, outbreaks depend on an urban human-mosquito-human cycle in which *Aedes* aegypti is usually the dominant vector.

### 9.2. Risk Factors

CHIKV transmission results from interacting climatic, environmental, population, and socioeconomic conditions. Temperature and precipitation affect vector development, survival, and viral replication; however, Mexican studies suggest that social and economic context may explain transmission intensity more strongly in some locations.

Poverty, unreliable water services, inadequate sanitation, crowded or substandard housing, rapid urbanization, and limited healthcare access all favor mosquito proliferation and sustained transmission. Seasonal increases in temperature and rainfall remain important because they accelerate vector abundance and viral circulation.

Among environmental variables, temperature and rainfall most consistently correlate with outbreak risk, whereas altitude and minimum precipitation generally contribute less to prediction. Effective surveillance should therefore combine climatic indicators with the social and environmental determinants that jointly shape transmission [59].

### 9.3. Prediction of Outbreaks

Reliable outbreak forecasting is difficult because routine surveillance may miss asymptomatic infections, depend on limited laboratory testing, receive delayed reports, and lose sensitivity during prolonged periods of monitoring [50].

Mexican spatiotemporal analyses have modeled dengue, Zika, and chikungunya jointly and have applied machine-learning approaches such as eXtreme Gradient Boosting (XGBoost). Predictors have included lagged case incidence, temperature, precipitation, vegetation or environmental suitability, vector distribution, population density, mobility proxies, and socioeconomic conditions. XGBoost can capture nonlinear interactions and may improve short-term geographic risk stratification compared with conventional regression; however, performance depends on complete and timely surveillance data, transportability across states, and prospective external validation [59,60,61,62,63,64,65,66,67].

For Mexico, forecasting should be linked operationally to routine epidemiologic, laboratory, entomologic, meteorologic, and genomic surveillance. Model outputs should trigger predefined actions—enhanced testing, vector investigation, community communication, and vaccine feasibility assessment—rather than remain solely descriptive. Current limitations include underdiagnosis, delayed reporting, changing case definitions, sparse serologic data, uneven vector sampling, and limited prospective validation during low-incidence periods.

### 9.4. Prevention and Control

Timely outbreak recognition requires surveillance that brings together clinical, laboratory, entomological, climatic, and epidemiological information. Rapid identification of suspected infections, coupled with monitoring of vectors and environmental conditions, enables earlier public health action to interrupt transmission [66,68].

Durable control remains difficult despite advances in vector interventions because mosquitoes disperse rapidly, insecticide resistance is increasing, people are highly mobile, and viruses are repeatedly introduced into susceptible populations. Modeling studies suggest that prompt case recognition and focused perifocal control can reduce outbreak magnitude when deployed early, particularly when coordinated with established dengue programs [56].

Integrated vector management remains the foundation of chikungunya prevention. Core activities include source reduction, sanitation, entomologic surveillance, risk-based monitoring, and habitat-suitability mapping. In Mexico, the mismatch between reported arboviral disease and the broad municipal distribution of *Aedes* mosquitoes argues for more complete surveillance and closer linkage of entomological and epidemiological information.

Sustained vector control also depends on community engagement. Programs should encourage removal of standing water, use of window and door screens, appropriate repellents and protective clothing, mosquito nets when indicated, and reduced exposure during peak biting periods. These practical actions remain central to integrated prevention [67].

PAHO and WHO advise countries to reinforce surveillance, laboratory diagnosis, clinical care, and coordinated vector-control programs for chikungunya and related arboviruses. Preparedness should include genomic monitoring to identify circulating genotypes and lineages and mutations that could affect vector adaptation or transmissibility. Reports should use the standardized categories West African, ECSA, Asian, and the ECSA-derived IOL [23].

Combining genomic data with epidemiologic observations, mobility patterns, and environmental indicators may improve risk forecasting and selection of preventive measures. The same integrated systems will be needed to measure the population impact of vaccination and refine immunization policy in endemic areas.

Overall, effective control of chikungunya requires a multidisciplinary approach combining surveillance, vector management, environmental interventions, community engagement, and vaccination. These complementary strategies provide the greatest opportunity to reduce transmission and mitigate the long-term health and socioeconomic consequences of CHIKV infection and strengthen preparedness for future outbreaks [23].

## 10. Vaccines Against Chikungunya

Recent advances in vaccine development have transformed the prevention of chikungunya virus (CHIKV) infection, culminating in the licensure of the first vaccines specifically designed to prevent this emerging arboviral disease. Several vaccine platforms—including live-attenuated, virus-like particle (VLP), viral-vector, inactivated, and nucleic acid-based technologies—have been investigated. Among these, live-attenuated and VLP-based vaccines have progressed furthest in clinical development and currently represent the most advanced preventive strategies.

### 10.1. Live-Attenuated Vaccine: Ixchiq^®^ (Valneva)

Ixchiq (VLA1553; Valneva) is a live-attenuated vaccine derived from the LR2006 OPY1 isolate of the East/Central/South African lineage. Attenuation was produced by deleting 61 amino acids from nsP3, thereby limiting replication while retaining immunogenicity. This engineered modification is genetically stable and permits a protective response after one dose [69].

In clinical development, a single dose produced seroconversion in approximately 99% of recipients. Neutralizing antibodies persisted in more than 96% at six months, and follow-up demonstrated protective levels for at least two years. Subsequent observations support persistence for four years or longer across age groups [70,71,72].

The overall safety profile has been considered acceptable. Headache, fever, muscle or joint pain, and transient fatigue are the adverse reactions reported most often, generally with mild or moderate intensity. Reactogenicity has been broadly similar to that of other licensed live-attenuated viral vaccines [4,73].

Ixchiq^®^ became the first licensed chikungunya vaccine for the prevention of chikungunya virus infection following approval by the U.S. Food and Drug Administration (FDA) in November 2023 for adults at increased risk of exposure. Subsequent regulatory approvals were granted in Canada, the United Kingdom, and the European Union [74,75,76,77,78].

Post-licensure pharmacovigilance identified rare but serious adverse events, concentrated mainly among adults aged 65 years or older and persons with multiple chronic conditions. Regulators responded differently as evidence evolved: the EMA temporarily restricted use in adults aged ≥65 years, then lifted the age restriction after review while retaining stronger warnings and recommending vaccination only when exposure risk is substantial; the U.S. FDA suspended the biologics license in August 2025, and the manufacturer subsequently discontinued U.S. distribution and requested voluntary license revocation. These actions should be interpreted through individualized and jurisdiction-specific risk–benefit assessment: both severe chikungunya and vaccine-associated adverse events are uncommon but clinically important, and the balance varies with age, comorbidity, destination, outbreak intensity, and availability of a non-replicating alternative [70,71,74,75,79,80,81].

Use during the 2025 outbreak on La Reunion substantially increased real-world experience: about 40,000 people received Ixchiq, the largest post-authorization deployment reported at that time. Follow-up from this campaign may clarify effectiveness, duration of protection, and longer-term safety in outbreak conditions [71,82].

### 10.2. Virus-like Particle Vaccine: Vimkunya^®^ (Bavarian Nordic)

A virus-like particle platform mimics the external architecture of CHIKV without carrying replicating viral genetic material. This design can elicit strong immune responses while avoiding replication, a combination that underlies its favorable safety profile.

The VLP candidate initially designated VRC-CHKVLP059-00-VP contains capsid, E3, E2, 6K, and E1 from the Senegal 37997 strain. These structural proteins assemble spontaneously into particles resembling native virions, stimulate potent antibody responses, and cannot reproduce in the recipient [83].

Clinical trials found strong immunogenicity and good tolerability. Reported reactions were predominantly mild or moderate, and neutralizing antibody responses were comparable with those generated by other advanced CHIKV vaccine approaches [76,77].

Bavarian Nordic markets the VLP vaccine as Vimkunya. FDA authorization was granted in 2025, followed by EMA authorization for persons 12 years of age or older. Its one-dose schedule, shared with Ixchiq, is operationally convenient for travelers and could support broader campaigns if future recommendations expand [72,84].

### 10.3. Future Perspectives

Two chikungunya vaccines have received approvals in one or more jurisdictions, but availability and regulatory status vary by country and continue to evolve. Current recommendations have generally prioritized travelers, laboratory workers, and other persons with substantial exposure risk rather than universal vaccination in endemic populations. This reflects incomplete evidence on clinical effectiveness, duration of protection, rare adverse events, cost-effectiveness, supply, and operational feasibility, rather than an absence of potential public health value.

The main characteristics and current safety considerations of the two chikungunya vaccines are summarized in Table 5.

Mexico still needs evidence to define priority groups, economic value, duration of immunity, booster needs, and integration with existing arbovirus programs. Older adults, people with comorbidities, and others at greater risk of severe or chronic disease may become important target groups as evidence and recommendations mature.

A large Brazilian pilot program represents an important step toward broader use. Valneva and Instituto Butantan are vaccinating approximately 500,000 adults aged 18–59 years outside an active outbreak, creating an opportunity to evaluate feasibility, performance, and public health impact under routine conditions [85].

Development continues beyond the two licensed products. mRNA, viral-vector, inactivated, and newer live-attenuated candidates remain under investigation. Trial results, pharmacovigilance, and real-world effectiveness studies will determine how prominently vaccination features in future integrated prevention programs [86,87].

### 10.4. Economic Burden and Value Assessment

Chikungunya generates direct costs from diagnostic testing, outpatient visits, hospitalization, rehabilitation, and prolonged rheumatologic care, as well as indirect costs from absenteeism, reduced productivity, caregiving, and disability. Regional estimates vary substantially by epidemic size, health-system prices, chronic disease frequency, and analytic perspective; Mexico-specific cost and cost-effectiveness evidence remains insufficient for a definitive national vaccination threshold.

Before introduction, Mexico should conduct budget-impact and cost-effectiveness analyses comparing outbreak-response vaccination, geographically targeted vaccination, and vaccination of high-risk occupational or clinical groups. Models should include underreporting, chronic arthralgia, quality-adjusted life-years, vaccine procurement and delivery, cold-chain and training costs, safety monitoring, and the opportunity cost of diverting resources from dengue and other immunization activities.

### 10.5. Chikungunya in the Mexican Immunization Program

Mexico can build on its national notifiable-disease surveillance, state public health laboratories, vector-control programs, and established vaccination infrastructure, but chikungunya vaccination is not currently positioned for immediate universal routine use. Key barriers include uncertain national disease burden and seroprevalence, underdiagnosis, variable laboratory capacity, absence of a stable procurement and financing pathway, evolving international guidance, product-specific safety considerations, and limited programmatic effectiveness data in endemic settings.

A staged policy pathway is therefore recommended: (1) strengthen case-based, laboratory, entomologic, climatic, and genomic surveillance; (2) define priority states and populations through burden and seroprevalence studies; (3) establish regulatory, procurement, cold-chain, training, communication, and pharmacovigilance requirements; (4) evaluate targeted demonstration projects with explicit effectiveness, safety, equity, and cost endpoints; and (5) consider broader implementation only after review by Mexican regulatory and immunization authorities. Operational coordination with dengue surveillance and vector-control activities is appropriate, but vaccine indications, contraindications, and product-specific schedules must remain distinct.

Brazilian implementation experience may inform logistics, acceptance, active safety surveillance, and effectiveness measurement, but transfer to Mexico will require adjustment for differences in epidemiology, regulatory status, population immunity, health-system organization, and financing. Policy should remain adaptable as WHO recommendations and post-marketing evidence mature.

## 11. Conclusions

Once limited geographically, CHIKV now represents a continuing global health concern. Climate change, urbanization, human movement, and the extensive range of *Aedes* vectors make recurrent transmission plausible in endemic and newly affected areas. Mexico’s largest epidemic occurred in 2014–2015, but competent vectors and suitable ecological conditions persist, leaving the country vulnerable to renewed outbreaks.

The impact of chikungunya extends well beyond the acute febrile episode. Persistent arthralgia and inflammatory arthritis can compromise mobility, daily function, and quality of life while generating healthcare and societal costs. These lasting effects support a transition from outbreak-driven responses to a more comprehensive prevention model [88].

The Immunization Committee of the Mexican Association of Pediatric Infectious Diseases considers vector control necessary but insufficient as Mexico’s sole long-term strategy. Environmental measures, entomologic surveillance, integrated vector management, and early outbreak recognition must continue, while effective vaccines offer an additional means of strengthening national preparedness.

Authorization of Ixchiq and Vimkunya has introduced a new phase in chikungunya prevention. Uncertainty remains regarding long-term effectiveness, post-marketing safety, protection duration, population prioritization, and implementation in endemic settings. Even so, vaccination can be considered within comprehensive planning when local epidemiology and regulatory guidance support its use.

On the basis of current evidence, the AMIP Immunization Committee proposes that Mexico reinforce chikungunya preparedness through a coordinated strategy comprising:

- Enhanced epidemiological, entomological, climatic, and genomic surveillance.
- Early laboratory confirmation and timely outbreak detection.
- Continuous evaluation of disease burden and chronic sequelae.
- Economic and operational evaluation of vaccine implementation in endemic settings.
- Prioritization of groups most vulnerable to severe disease, chronic sequelae, or disability.
- Collaborative studies capable of producing Mexican evidence for future immunization decisions.

Future recommendations should be developed jointly by public health agencies, academic centers, professional societies, and regulatory authorities. Policy should be revised as new information emerges on effectiveness, safety, persistence of protection, and programmatic experience.

The Committee concludes that chikungunya should be treated as a sustained health priority for Mexico and the wider Americas, rather than only as an episodic emerging infection. Coordinating vaccination with surveillance, vector interventions, community participation, and clinical preparedness offers the strongest prospect of limiting transmission, chronic disability, and future health and economic losses.

## 12. Limitations

This paper is limited by its reliance on published studies and official reports, which are affected by publication bias, variation in study methods, and epidemiologic information that continues to change. Because the objective was expert guidance for Mexico rather than a systematic evidence synthesis, the authors did not undertake a formal risk-of-bias evaluation or systematic-review methodology.

## Figures and Tables

**Table 1:** Laboratory diagnosis of chikungunya virus infection according to disease stage, specimen, and method.

Disease Stage/Timing	Method	Preferred Specimen	Advantages	Limitations/Interpretation
Acute, days 0–7 (highest yield usually ≤5 days)	RT-qPCR/RT-PCR	Serum or plasma; whole blood where validated	High specificity; confirms current infection; enables genomic analysis	Yield falls as viremia wanes; requires molecular laboratory capacity
Acute, generally days 1–7	Viral antigen assay (only where locally validated)	Serum or plasma	Potentially rapid and useful before seroconversion	Performance varies by assay; negative results do not exclude infection
Acute, usually days 0–7	Viral isolation	Serum or plasma	Demonstrates viable virus; useful for research and characterization	Slow; low sensitivity; requires specialized high-containment laboratory
Late acute/convalescent, from approximately day 5–7	CHIKV IgM by MAC-ELISA or validated serologic assay	Serum; paired samples when needed	Useful after viremia declines	Poor sensitivity if obtained too early; persistence and alphavirus cross-reactivity may complicate interpretation; confirm when epidemiologically indicated
Convalescent/retrospective, paired samples 2–3 weeks apart	CHIKV IgG seroconversion or ≥4-fold rise in titer	Paired acute and convalescent serum	Supports recent infection and retrospective confirmation	A single IgG-positive sample generally indicates prior exposure and does not date infection

Note: Timing is approximate and must be interpreted with symptom onset, local assay validation, vaccination history, and co-circulation of other alphaviruses. RT-qPCR/RT-PCR is preferred during early viremia; serology becomes more informative after approximately day 5–7 [49,51,52].

**Table 2:** Diagnostic performance of currently available laboratory tests for the diagnosis of chikungunya virus (CHIKV) infection.

Diagnostic Test	Sensitivity (%)	Specificity (%)	PPV (%)	NPV (%)
RT-qPCR	88.5	100	100	97.5
Viral isolation	15–50	100	100	63.0
Standard Diagnostics CHIKV IgM ELISA	3.9	92.5	10.0	81.6
Novatec CHIKV IgM Capture ELISA	76.9	91.9	100	97.5
Novatec CHIKV IgG ELISA	80.0	100	100	95.6

Abbreviations: PPV, positive predictive value; NPV, negative predictive value; RT-qPCR, reverse-transcription quantitative polymerase chain reaction; IgM, immunoglobulin M; IgG, immunoglobulin G; ELISA, enzyme-linked immunosorbent assay. The 3.9% sensitivity reported for the Standard Diagnostics CHIKV IgM ELISA refers to acute specimens collected at a median of approximately 5 days of illness (IQR 3–7), before antibody assays achieve their optimal yield; the same study reported 84% sensitivity in convalescent specimens. It should not be interpreted as the general sensitivity of IgM ELISA across all disease stages. Exact confidence intervals were not consistently available for every value in the source tables; therefore, point estimates are retained, and their study-specific nature is emphasized [52,53].

**Table 3:** Clinical and diagnostic features that help distinguish chikungunya, dengue, and Zika virus infection.

Feature	Chikungunya	Dengue	Zika
Fever	Usually abrupt and high	Usually high	Often absent or low-grade
Arthralgia/arthritis	Prominent, often severe and disabling; may persist	Usually less prominent	Common, generally milder
Myalgia/headache	Common	Often prominent; retro-orbital pain may occur	Usually mild
Rash	Common	Common	Common, often pruritic
Bleeding/shock	Rare	Characteristic warning sign in severe dengue	Uncommon
Key complications	Chronic inflammatory rheumatism; neurologic/cardiac disease	Plasma leakage, severe bleeding, organ impairment	Congenital Zika syndrome; neurologic complications
Preferred early confirmation	CHIKV RT-PCR	DENV NAAT and/or NS1 antigen	ZIKV NAAT according to specimen and timing
Important caution	Serology can cross-react with other alphaviruses	Avoid NSAIDs until dengue is excluded	Pregnancy requires specific diagnostic and fetal assessment pathways

**Table 4:** Clinical Pathway from Symptom Onset to Disposition.

Step	Clinical Action
1. Initial assessment	Document day of illness, exposure/travel, pregnancy, age, comorbidities, hydration, hemodynamic status, bleeding, neurologic or cardiopulmonary findings.
2. Differential diagnosis	Consider dengue, Zika, malaria, leptospirosis, rickettsiosis, measles/rubella, and bacterial sepsis according to epidemiology.
3. Testing strategy	Days 0–7: prioritize CHIKV RT-PCR and concurrent dengue testing. After day 5–7: add validated CHIKV IgM; obtain paired IgG when clarification is needed.
4. Outpatient pathway	Stable patient without warning signs: hydration, acetaminophen, mosquito-bite avoidance during the first week, return precautions, and reassessment if symptoms persist.
5. Admit/urgent referral	Hemodynamic instability, significant bleeding, organ dysfunction, altered mental status, respiratory compromise, severe dehydration, uncontrolled pain, pregnancy near delivery, neonate/young infant, or decompensated comorbidity.
6. Follow-up/specialist referral	Persistent inflammatory joint symptoms >3 months, functional limitation, neurologic sequelae, or diagnostic uncertainty: rheumatology, neurology, rehabilitation, or infectious diseases as appropriate.

**Table 5:** Comparison of the principal characteristics and current safety considerations of licensed chikungunya vaccines.

Characteristic	Vimkunya^®^	Ixchiq^®^
Platform	Virus-like particle (VLP), non-replicating	Live-attenuated, replication-competent
Manufacturer	Bavarian Nordic	Valneva SE
Schedule/route	Single 0.8 mL intramuscular dose	Single intramuscular dose
Age indication	≥12 years in the United States and European Union	Adults ≥18 years in authorizing jurisdictions; current status and recommendations vary by country *
Initial approvals	FDA: February 2025; EMA: 2025	FDA: November 2023; subsequently EU, Canada, and UK
Main advantage	Non-replicating platform; favorable trial safety profile	High seroresponse after one dose
Main considerations	Limited real-world effectiveness and long-term protection data; immune response may be reduced in immunocompromised persons **	Rare serious post-licensure events occurred mainly in older adults with comorbidities; regulatory actions differed by jurisdiction; individualized risk–benefit assessment and pharmacovigilance are required *

* Regulatory status is time- and jurisdiction-specific. The FDA suspended the U.S. biologics license in August 2025; Valneva later discontinued U.S. manufacture/distribution and requested voluntary revocation. EMA lifted its temporary ≥ 65-year restriction after review but retained enhanced warnings and advised use only when exposure risk is substantial. Canadian guidance recommends against use in persons ≥ 65 years. Consult current national guidance [74,75,79,80,81]. ** A non-replicating platform is not equivalent to proven safety or effectiveness in every immunocompromised population; clinical decision-making should follow the current product information and national recommendations.

## Data Availability

No new data were created or analyzed in this study. Data sharing is not applicable to this article.

## References

[1] Rama K., de Roo A.M., Louwsma T., Hofstra H.S., Gurgel do Amaral G.S., Vondeling G.T., Postma M.J., Freriks R.D. (2024). Clinical outcomes of chikungunya: A systematic literature review and meta-analysis. PLoS Negl. Trop. Dis..

[2] Kang H., Auzenbergs M., Clapham H., Maure C., Kim J.H., Salje H., Taylor C.G., Lim A., Clark A., Edmunds W.J. (2024). Chikungunya seroprevalence, force of infection, and prevalence of chronic disability after infection in endemic and epidemic settings: A systematic review, meta-analysis, and modelling study. Lancet Infect. Dis..

[3] World Health Organization (2024). Chikungunya Fact Sheet.

[4] Schneider M., Narciso-Abraham M., Hadl S., McMahon R., Toepfer S., Fuchs U., Hochreiter R., Bitzer A., Kosulin K., Larcher-Senn J. (2023). Safety and immunogenicity of a single-shot live-attenuated chikungunya vaccine: A double-blind, multicentre, randomised, placebo-controlled, phase 3 trial. Lancet.

[5] Pan American Health Organization (2023). Chikungunya: Epidemiological Update in the Americas.

[6] Centers for Disease Control and Prevention (2026). Chikungunya Vaccine: Clinical Guidance for Healthcare Providers.

[7] U.S. Food and Drug Administration (2023). FDA Approves First Vaccine to Prevent Disease Caused by Chikungunya Virus.

[8] Burt F.J., Rolph M.S., Rulli N.E., Mahalingam S., Heise M.T. (2012). Chikungunya: A re-emerging virus. Lancet.

[9] Strauss J.H., Strauss E.G. (1994). The alphaviruses: Gene expression, replication, and evolution. Microbiol. Rev..

[10] Agarwal R., Chang J., Côrtes F.H., Ha C., Villalpando J., Castillo I.N., Gálvez R.I., Grifoni A., Sette A., Romero-Vivas C.M. (2025). Chikungunya virus-specific CD4+ T cells are associated with chronic chikungunya viral arthritic disease in humans. Cell Rep. Med..

[11] Barth B.U., Suomalainen M., Liljeström P., Garoff H. (1992). Alphavirus assembly and entry: Role of the cytoplasmic tail of the E1 spike subunit. J. Virol..

[12] Weaver S.C., Lecuit M. (2015). Chikungunya virus and the global spread of a mosquito-borne disease. N. Engl. J. Med..

[13] Zhang R., Kim A.S., Fox J.M., Nair S., Basore K., Klimstra W.B., Rimkunas R., Fong R.H., Lin H., Poddar S. (2018). Mxra8 is a receptor for multiple arthritogenic alphaviruses. Nature.

[14] Hamrahjoo M., Shams F., Saadat N., Marhamati S., Teimoori A. (2026). Overview of chikungunya virus pathogenesis, genome variation, epidemiology, and control. Virus Res..

[15] Rudolph K.E., Lessler J., Moloney R.M., Kmush B., Cummings D.A. (2014). Incubation periods of mosquito-borne viral infections: A systematic review. Am. J. Trop. Med. Hyg..

[16] Gasque P., Couderc T., Lecuit M., Roques P., Ng L.F. (2015). Chikungunya virus pathogenesis and immunity. Vector-Borne Zoonotic Dis..

[17] Sourisseau M., Schilte C., Casartelli N., Trouillet C., Guivel-Benhassine F., Rudnicka D., Sol-Foulon N., Le Roux K., Prevost M.-C., Fsihi H. (2007). Chikungunya virus infection of lymphoid tissue and persistence. J. Virol..

[18] Tian J., Li Y., Zhao Y., Tao X. (2026). Visualized analysis of core themes and emerging frontiers in global chikungunya virus studies. Front. Microbiol..

[19] Oliveira-Neto J.T., Souza J.P., Rodrigues D., Machado M.R., Alves J.V., Barros P.R., Bressan A.F., Silva J.F., Costa T.J., Costa R.M. (2024). Acute chikungunya infection induces vascular dysfunction by directly disrupting redox signaling in endothelial cells. Cells.

[20] de Souza W.M., Fumagalli M.J., de Lima S.T.S., Parise P.L., Carvalho D.C.M., Hernandez C., de Jesus R., Delafiori J., Candido D.S., Carregari V.C. (2024). Pathophysiology of chikungunya virus infection associated with fatal outcomes. Cell Host Microbe.

[21] Thiberville S.D., Moyen N., Dupuis-Maguiraga L., Nougairede A., Gould E.A., Roques P., de Lamballerie X. (2013). Chikungunya fever: Epidemiology, clinical syndrome, pathogenesis and therapy. Antivir. Res..

[22] Powers A.M., Logue C.H. (2007). Changing patterns of chikungunya virus: Re-emergence of a zoonotic arbovirus. J. Gen. Virol..

[23] Pan American Health Organization, World Health Organization (2025). Epidemiological Alert: Chikungunya and Oropouche in the Americas Region, 28 August 2025.

[24] Nsoesie E.O., Kraemer M.U.G., Golding N., Pigott D.M., Brady O.J., Moyes C.L., Johansson M.A., Gething P.W., Velayudhan R., Khan K. (2016). Global distribution and environmental suitability for chikungunya virus, 1952 to 2015. Eurosurveillance.

[25] Sergon K., Njuguna C., Kalani R., Ofula V., Onyango C., Konongoi L.S., Bedno S., Burke H., Dumilla A.M., Konde J. (2008). Seroprevalence of Chikungunya virus infection on Lamu Island, Kenya, October 2004. Am. J. Trop. Med. Hyg..

[26] Angelini R., Finarelli A.C., Angelini P., Po C., Petropulacos K., Macini P., Fiorentini C., Fortuna C., Venturi G., Romi R. (2007). An outbreak of chikungunya fever in the province of Ravenna, Italy. Eurosurveillance.

[27] World Health Organization (2025). Chikungunya Epidemiology Update—June 2025.

[28] Gutierrez B., da Silva Candido D., Bajaj S., Rodriguez Maldonado A.P., Ayala F.G., Rodriguez M.d.l.L.T., Rodriguez A.A., Arámbula C.W., González E.R., Martínez I.L. (2023). Convergent trends and spatiotemporal patterns of Aedes-borne arboviruses in Mexico and Central America. PLoS Negl. Trop. Dis..

[29] Gonzalez-Perez A.L., Vazquez A., de Ory F., Negredo A., Plante K.S., Plante J.S., Palermo P.M., Watts D., Paz Sanchez-Seco M., Weaver S.C. (2024). Outbreak of chikungunya fever in the Central Valley of Chiapas, Mexico. medRxiv.

[30] Nunez-Avellaneda D., Tangudu C., Barrios-Palacios J., Salazar M.I., Machain-Williams C., Cisneros-Pano J., McKeen L.A., Blitvich B.J. (2021). Chikungunya in Guerrero, Mexico, 2019 and evidence of gross underreporting in the region. Am. J. Trop. Med. Hyg..

[31] Staples J.E., Breiman R.F., Powers A.M. (2009). Chikungunya fever: An epidemiological review of a re-emerging infectious disease. Clin. Infect. Dis..

[32] Queyriaux B., Simon F., Grandadam M., Michel R., Tolou H. (2008). Clinical burden of chikungunya virus infection. Lancet Infect. Dis..

[33] Burt F.J., Chen W., Miner J.J., Lenschow D.J., Merits A., Schnettler E., Kohl A., Rudd P.A., Taylor A., Herrero L. (2017). Chikungunya virus: An update on the biology and pathogenesis of this emerging pathogen. Lancet Infect. Dis..

[34] Simon F., Javelle E., Oliver M., Leparc-Goffart I., Marimoutou C. (2011). Chikungunya virus infection. Curr. Infect. Dis. Rep..

[35] Borgherini G., Poubeau P., Staikowsky F., Lory M., Le Moullec N., Becquart J.P., Wengling C., Michault A., Paganin F. (2007). Outbreak of chikungunya on Reunion Island: Early clinical and laboratory features in 157 adult patients. Clin. Infect. Dis..

[36] Gérardin P., Fianu A., Michault A., Mussard C., Boussaid K., Rollot O., Grivard P., Kassab S., Bouquillard E., Borgherini G. (2013). Predictors of chikungunya rheumatism: A prognostic survey ancillary to the TELECHIK cohort study. Arthritis Res. Ther..

[37] Couderc T., Chrétien F., Schilte C., Disson O., Brigitte M., Guivel-Benhassine F., Touret Y., Barau G., Cayet N., Schuffenecker I. (2008). A mouse model for chikungunya: Young age and inefficient type-I interferon signaling are risk factors for severe disease. PLoS Pathog..

[38] Economopoulou A., Dominguez M., Helynck B., Sissoko D., Wichmann O., Quenel P., Germonneau P., Quatresous I. (2009). Atypical chikungunya virus infections: Clinical manifestations, mortality and risk factors for severe disease during the 2005–2006 outbreak on Réunion. Epidemiol. Infect..

[39] Gérardin P., Barau G., Michault A., Bintner M., Randrianaivo H., Choker G., Lenglet Y., Touret Y., Bouveret A., Grivard P. (2008). Multidisciplinary prospective study of mother-to-child chikungunya virus infections on the island of Réunion. PLoS Med..

[40] Hoarau J.J., Jaffar Bandjee M.C., Trotot P.K., Das T., Li-Pat-Yuen G., Dassa B., Denizot M., Guichard E., Ribera A., Henni T. (2010). Persistent chronic inflammation and infection by chikungunya arthritogenic alphavirus in spite of a robust host immune response. J. Immunol..

[41] Javelle E., Ribera A., Degasne I., Gaüzère B.A., Marimoutou C., Simon F. (2015). Specific management of post-chikungunya rheumatic disorders: A retrospective study of 159 cases in Réunion Island. PLoS Negl. Trop. Dis..

[42] Kelvin A.A., Banner D., Silvi G., Moro M.L., Spataro N., Gaibani P., Cavrini F., Pierro A., Rossini G., Cameron M.J. (2011). Inflammatory cytokine expression is associated with chikungunya virus resolution and symptom severity. PLoS Negl. Trop. Dis..

[43] Schilte C., Couderc T., Chrétien F., Sourisseau M., Gangneux N., Guivel-Benhassine F., Kraxner A., Tschopp J., Higgs S., Michault A. (2010). Type I IFN controls chikungunya virus via its action on nonhematopoietic cells. J. Exp. Med..

[44] Kam Y.W., Simarmata D., Chow A., Her Z., Teng T.S., Ong E.K., Rénia L., Leo Y.-S., Ng L.F.P. (2012). Early appearance of neutralizing IgG3 antibodies is associated with chikungunya virus clearance and long-term clinical protection. J. Infect. Dis..

[45] Lum F.M., Teo T.H., Lee W.W., Kam Y.W., Rénia L., Ng L.F. (2013). An essential role of antibodies in the control of chikungunya virus infection. J. Immunol..

[46] Ng L.F., Chow A., Sun Y.J., Kwek D.J., Lim P.L., Dimatatac F., Ng L.-C., Ooi E.-E., Choo K.-H., Her Z. (2009). IL-1β, IL-6, and RANTES as biomarkers of chikungunya severity. PLoS ONE.

[47] Galán-Huerta K.A., Rivas-Estilla A.M., Fernández-Salas I., Farfan-Ale J.A., Ramos-Jiménez J. (2015). Chikungunya virus: A general overview. Med. Univ..

[48] Suryavanshi K.T., Chakraborty A. (2023). Investigation of chikungunya virus genotype at tertiary care center in Western Maharashtra, India. J. Vector Borne Dis..

[49] Soto-Garita C., Carrera J., López-Vergès S., Corrales-Aguilar E. (2018). Advances in clinical diagnosis and management of chikungunya virus infection. Curr. Treat. Options Infect. Dis..

[50] (2017). Chikungunya virus: Clinical aspects and treatment—A review. Mem. Inst. Oswaldo Cruz..

[51] Khan T. (2025). Diagnosis of chikungunya infection: A narrative review. Sir Salimullah Med. Coll. J..

[52] Andrew A., Navien T., Yeoh T., Citartan M., Mangantig E., Sum M., Ch’ng E.S., Tang T.-H. (2022). Diagnostic accuracy of serological tests for the diagnosis of chikungunya virus infection: A systematic review and meta-analysis. PLoS Negl. Trop. Dis..

[53] Blacksell S.D., Tanganuchitcharnchai A., Jarman R.G., Gibbons R.V., Paris D.H., Bailey M.S., Day N.P.J., Premaratna R., Lalloo D.G., de Silva H.J. (2011). Poor diagnostic accuracy of commercial antibody-based assays for the diagnosis of acute chikungunya infection. Clin. Vaccine Immunol..

[54] Freppel W., Silva L., Stapleford K., Herrero L. (2024). Pathogenicity and virulence of chikungunya virus. Virulence.

[55] Webb E., Michelen M., Rigby I., Dagens A., Dahmash D., Cheng V., Joseph R., Lipworth S., Harriss E., Cai E. (2022). An evaluation of global chikungunya clinical management guidelines: A systematic review. EClinicalMedicine.

[56] Ndeffo-Mbah M., Durham D., Skrip L., Nsoesie E.O., Brownstein J.S., Fish D., Galvani A.P. (2016). Evaluating the effectiveness of localized control strategies to curtail chikungunya. Sci. Rep..

[57] Monteiro V., Navegantes-Lima K., Lemos A., Silva G., Souza Gomes R., Reis J.F., Junior L.R., Da Silva O.S., Romão P.R.T., Monteiro M.C. (2019). *Aedes*–chikungunya virus interaction: Key role of vector midguts microbiota and its saliva in the host infection. Front. Microbiol..

[58] Morsy T., Elgohary M., Sallam T., Morsy A. (2023). Chikungunya virus versus Aedes-borne other viruses: A review article. J. Egypt. Soc. Parasitol..

[59] Dong B., Khan L., Smith M., Trevino J., Zhao B., Hamer G.L., Lopez-Lemus U.A., Molina A.A., Lubinda J., Nguyen U.-S.D.T. (2022). Spatio-temporal dynamics of three diseases caused by Aedes-borne arboviruses in Mexico. Commun. Med..

[60] Roques P., Thiberville S.D., Dupuis-Maguiraga L., Lum F.M., Labadie K., Martinon F., Gras G., Jaffar-Bandjee M.-C., Gasque P., Simon F. (2017). Paradoxical effect of antibodies in chikungunya virus disease. Expert Rev. Anti Infect. Ther..

[61] Teo T.H., Her Z., Tan J.J., Lum F.M., Lee W.W., Chan Y.H., Ong R.Y., Kam Y.W., Leparc-Goffart I., Gallian P. (2015). Caribbean and Latin American outbreak strains of chikungunya virus differ in virulence in a mouse model. PLoS Negl. Trop. Dis..

[62] Silva L.A., Dermody T.S. (2017). Chikungunya virus: Epidemiology, replication, disease mechanisms, and prospective intervention strategies. J. Clin. Investig..

[63] Morrison T.E. (2014). Reemergence of chikungunya virus. J. Virol..

[64] Wahid B., Ali A., Rafique S., Idrees M. (2017). Global expansion of chikungunya virus: Mapping the 21st-century epidemic. Int. J. Infect. Dis..

[65] Vairo F., Haider N., Kock R., Ntoumi F., Ippolito G., Zumla A. (2019). Chikungunya: Epidemiology, pathogenesis, clinical features, management, and prevention. Infect. Dis. Clin. N. Am..

[66] Ooi E.E., Goh K.T., Gubler D.J. (2006). Dengue prevention and 35 years of vector control in Singapore. Emerg. Infect. Dis..

[67] Lubinda J., Treviño C.J., Walsh M.R., Moore A.J., Hanafi-Bojd A.A., Akgun S., Zhao B., Barro A.S., Begum M.M., Jamal H. (2019). Environmental suitability for *Aedes aegypti* and *Aedes albopictus* and the spatial distribution of major arboviral infections in Mexico. Parasite Epidemiol. Control.

[68] Escobar L.E., Qiao H., Peterson A.T. (2016). Forecasting Chikungunya spread in the Americas via data-driven empirical approaches. Parasites Vectors.

[69] Chen L.H., Fritzer A., Hochreiter R., Dubischar K., Meyer S. (2024). From bench to clinic: The development of VLA1553/IXCHIQ, a live-attenuated chikungunya vaccine. J. Travel Med..

[70] The Lancet Infectious Diseases (2025). More data needed on the FDA’s decision to suspend Ixchiq. Lancet Infect. Dis..

[71] Monaco Times (2025). Chikungunya Epidemic in La Réunion Marks Twelve Deaths in 2025.

[72] Centers for Disease Control and Prevention (2026). Chikungunya Vaccine Information for Healthcare Providers.

[73] Maurer G., Buerger V., Larcher-Senn J., Erlsbacher F., Meyer S., Eder-Lingelbach S., Jaramillo J.C. (2025). Comprehensive assessment of reactogenicity and safety of the live-attenuated chikungunya vaccine (IXCHIQ^®^). Vaccines.

[74] European Medicines Agency (2025). Ixchiq: Temporary Restriction on Vaccinating People 65 Years and Older to Be Lifted. https://www.ema.europa.eu/en/news/ixchiq-temporary-restriction-vaccinating-people-65-years-older-be-lifted.

[75] Public Health Agency of Canada, Committee to Advise on Tropical Medicine and Travel (CATMAT) (2025). Recommendations for the Use of Chikungunya Live Attenuated Vaccine (IXCHIQ).

[76] Richardson J.S., Anderson D.M., Mendy J., Tindale L.C., Muhammad S., Loreth T., Tredo S.R., Warfield K.L., Ramanathan R., Caso J.T. (2025). CBSI-CV-317-004 Study Group. Chikungunya virus virus-like particle vaccine safety and immunogenicity in adolescents and adults in the USA: A phase 3, randomised, double-blind, placebo-controlled trial. Lancet.

[77] Tindale L.C., Richardson J.S., Anderson D.M., Mendy J., Muhammad S., Loreth T., Tredo S.R., Ramanathan R., A Jenkins V., EBSI-CV-317-005 Study Group (2025). Chikungunya virus virus-like particle vaccine safety and immunogenicity in adults older than 65 years: A phase 3, randomised, double-blind, placebo-controlled trial. Lancet.

[78] Medicines and Healthcare Products Regulatory Agency (2025). IXCHIQ Vaccine Approved to Protect Adults Against Chikungunya.

[79] European Medicines Agency (2025). Ixchiq-Referral.

[80] U.S. Food and Drug Administration (2025). FDA Update on the Safety of Ixchiq (Chikungunya Vaccine, Live): FDA Safety Communication.

[81] U.S. Food and Drug Administration (2025). FDA Update on the Safety of IXCHIQ (Chikungunya Vaccine, Live): FDA Suspends Biologics License. https://www.fda.gov/safety/medical-product-safety-information/fda-update-safety-ixchiq-chikungunya-vaccine-live-fda-suspends-biologics-license-fda-safety.

[82] Outbreak News Today (2025). Valneva to Provide 40,000 IXCHIQ Chikungunya Vaccine Doses.

[83] Ahola T., Couderc T., Ng L.F., Hallengärd D., Powers A., Lecuit M., Esteban M., Merits A., Roques P., Liljeström P. (2015). Therapeutics and vaccines against chikungunya virus. Vector Borne Zoonotic Dis..

[84] European Medicines Agency (2025). Vimkunya.

[85] Valneva S.E. (2026). Valneva and Instituto Butantan Announce Initiation of a Pilot Vaccination Campaign in Brazil with Single-Shot Chikungunya Vaccine IXCHIQ^®^.

[86] Teo T.H., Lum F.M., Lee W.W., Ng L.F. (2012). Mouse models for chikungunya virus: Deciphering immune mechanisms responsible for disease and protection. Immunol. Res..

[87] Teng T.S., Foo S.S., Simamarta D., Lum F.M., Teo T.H., Lulla A., Yeo N.K., Koh E.G., Chow A., Leo Y.-S. (2012). Viperin restricts chikungunya virus replication and pathology. J. Clin. Investig..

[88] Chow A., Her Z., Ong E.K., Chen J.M., Dimatatac F., Kwek D.J., Barkham T., Yang H., Rénia L., Leo Y.-S. (2011). Persistent arthralgia induced by chikungunya virus infection is associated with interleukin-6 and granulocyte macrophage colony-stimulating factor. J. Infect. Dis..

